# Silencing lncRNA FOXD2-AS1 inhibits proliferation, migration, invasion and drug resistance of drug-resistant glioma cells and promotes their apoptosis via microRNA-98-5p/CPEB4 axis

**DOI:** 10.18632/aging.102455

**Published:** 2019-11-26

**Authors:** Naibing Gu, Xinlai Wang, Zhengli Di, Jing Xiong, Yue Ma, Yu’e Yan, Yihua Qian, Quanzeng Zhang, Jia Yu

**Affiliations:** 1 Department of Human Anatomy, Histology and Embryology, School of Basic Medical Sciences, Xi’an Jiaotong University Health Science Center, Xi’an 710061, China; 2 Department of Neurology, Xi’an Central Hospital, Xi’an Jiaotong University School of Medicine, Xi’an 710003, China; 3 Department of Neurosurgery, Tangdu Hospital, Air Force Military Medical University, Xi’an 710038, China

**Keywords:** lncRNA FOXD2-AS1, microRNA-98-5p, CPEB4, glioma, temozolomide, drug resistance

## Abstract

Objective: This study was conducted to elucidate the long non-coding RNA FOXD2-AS1 (lncRNA FOXD2-AS1) expression in glioma and its mechanism on the biological features of glioma cells and the drug resistance of temozolomide (TMZ).

Results: Highly expressed FOXD2-AS1 was found in glioma. There was more powerful chemotherapeutic resistance of TMZ resistant cell lines than that of the parent cell lines. Silence of FOXD2-AS1 suppressed proliferation and drug resistance and promoted apoptosis of drug-resistant glioma cells. Overexpressed FOXD2-AS1 presented an opposite trend. FOXD2-AS1 could be used as a competing endogenous RNA to adsorb miR-98-5p, thereby up-regulating CPEB4.

Conclusion: Our study suggests that down-regulated FOXD2-AS1 repressed invasion, proliferation, migration and drug resistance of drug-resistant glioma cells while stimulating their apoptosis via increasing miR-98-5p and inhibiting CPEB4 expression.

Methods: FOXD2-AS1, microRNA-98-5p (miR-98-5p) and cytoplasmic polyadenylation element binding (CPEB4) expression in glioma tissues were tested. Expression of E-cadherin, N-cadherin and Vimentin in glioma cells were explored. A series of assays were conducted to detect the function of FOXD2-AS1 in migration, proliferation, apoptosis, and invasion of glioma cells. Changes in drug-resistance of cells under TMZ treatment were examined, and tumor formation in nude mice was performed to test the changes of drug resistance *in vivo*.

## INTRODUCTION

Glioma is the most common type of primary intracranial tumor, occupying 81% of malignant brain tumors, and it has high mortality and morbidity [1]. The highly invasive and infiltrative features of glioma determine its incurability and mortality, and it is special in uncontrollable growth due to unrestricted proliferation of tumor cells [2, 3]. Glioblastoma (GBM) is the most malignant glioma subtype in adults; the survival time of patients with GBM is only around 15 months, making GBM one of the most invasive cancers [4]. Temozolomide (TMZ), a new oral imidazotetrazinone methylating agent, has a schedule-dependent antitumor activity in all types of late cancers, including malignant gliomas [5]. TMZ-based chemotherapy is a typical strategy for treatment of glioma, while chemo-resistance acts as a main therapeutic challenge [6]. TMZ is the most frequently used chemotherapy for GBM. However, absence of obvious improvement of patient survival, developing drug resistance in recurrent tumor, and scarce alternative chemotherapy drugs are the main stumbling blocks for TMZ in glioma treatment [7]. Although great achievements have been made in multimodality treatments including radiotherapy, chemotherapy, and surgical resection, the poor prognosis of GBM has not improved for more than three decades [8]. With glioma’s unclear diagnosis, poor prognosis, and limited treatment methods, it is urgent to seek new therapeutic targets to improve the prognosis of the disease.

Long non-coding RNA FOXD2-AS1 (lncRNA FOXD2-AS1) is a newly found lncRNA which has been found to be highly expressed in various tumors [9, 10] LncRNA FOXD2-AS1 was overexpressed in glioma tissues and cells and showed poor prognosis in patients with glioma [10]. It is reported that lncRNA FOXD3-AS1 has been involved in glioma for its oncogenic nature [11]. In recent years, microRNAs (miRNAs) have been found to be involved in the progression of all kinds of cancers and regarded as novel targets for anticancer therapies [12]. As previously reported, miR-98 is down-regulated in glioma tissues and glioma cell lines relative to their normal counterparts [13, 14]. A study has suggested that miR-98 overexpression contributed to suppressed migration and invasion of glioma cells, but had no influence on the cell viability [15]. Some miRNAs promote or restrain tumor metastasis or invasion through upregulating or downregulating metastasis-related genes, offering potential therapeutic targets on anti-metastasis strategy [16]. Cytoplasmic polyadenylation element binding (CPEB4) is a RNA binding protein and a part of the CPEB family, and increased CPEB4 expression is implicated in migration, tumor growth, vascularization, metastasis and invasion [17]. Evidence has demonstrated that CPEB4 is highly expressed in human glioma and the up-regulation of CPEB4 protein is clearly related to advanced World Health Organization (WHO) grade [18, 19]. As a result, this study intends to figure out the lncRNA FOXD2-AS1 expression in glioma and its function on glioma cell biological behaviors and drug resistance of TMZ.

## RESULTS

### Highly expressed FOXD2-AS1 is found in glioma

**Figure 1:**
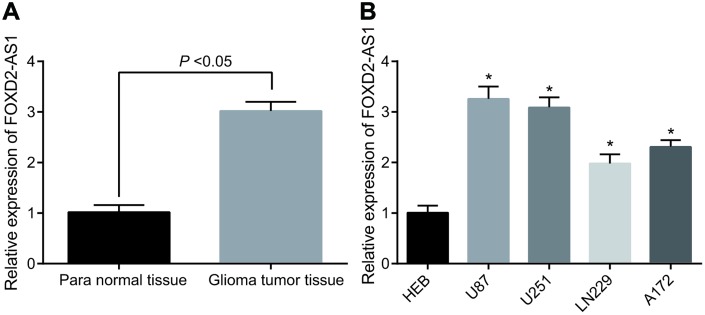
**Highly expressed FOXD2-AS1 is found in glioma.** (**A**) The expression level of FOXD2-AS1 in glioma tumor tissues and corresponding para normal tissues was detected by RT-qPCR (N = 68); (**B**) RT-qPCR was used to detect the expression of FOXD2-AS1 in human normal glial brain cell line HEB and 4 human glioma cell lines. * *P* < 0.05 vs human normal glial brain cell line HEB. The data were all measurement data, represented by mean ± standard deviation. The comparison between the two groups was statistically analyzed by independent sample t test, and one-way ANOVA was used in comparisons among multiple groups, and Tukey’s post-hoc test was performed after ANOVA. The experiment was repeated three times.

The expression level of FOXD2-AS1 was detected in 86 cases of glioma tumor tissues and their para normal tissues. The results of RT-qPCR manifested that FOXD2-AS1 expression in glioma tumor tissues was apparently elevated compared with that in the para normal tissues (*P* < 0.05) (Figure 1A). The relationship between FOXD2-AS1 expression and the clinicopathological characteristics of glioma patients was further analyzed, and it was found that the expression level of FOXD2-AS1 was not associated with the gender, age and histological type of patients (all *P* > 0.05), but related to tumor diameter and WHO classification, lymph node metastasis and TMZ drug resistance (all *P* < 0.05) (Table 1). The expression of FOXD2-AS1 in human normal glial brain cell line HEB and human glioma cell line (U87, U251, LN229, A172) were also detected by RT-qPCR. The results suggested that (Figure 1B) there were varying degrees of higher expression of FOXD2-AS1 in 4 kinds of glioma cells in contrast with HEB cells (all *P* < 0.05), of which FOXD2-AS1 was obviously expressed in the U87 and U251 cell lines, which were chosen for subsequent experiments.

**Table 1:** Correlation of clinicopathological characteristics between FOXD2-AS1 and glioma patients.

**Clinicopathologic data**	**Case (n)**	**FOXD2-AS1 expression**	***P***
Age (years)			0.109
< 60	45	2.97 ± 0.29	
≥ 60	41	2.88 ± 0.43	
Gender			0.331
Male	52	2.97 ± 0.22	
Female	34	3.01 ± 0.31	
Tumor size (cm)			< 0.001
< 3	57	1.66 ± 0.40	
≥ 3	29	2.97 ± 0.24	
Histological type			0.235
AT	46	2.95 ± 0.32	
OT	19	2.94 ± 0.26	
Other	21	3.01 ± 0.29	
WHO classification			< 0.001
I+II	62	1.69 ± 0.20	
III+IV	24	2.97 ± 0.26	
Lymph node metastasis			< 0.001
Yes	60	1.58 ± 0.13	
No	26	2.80±0.25	
TMZ drug resistance			< 0.001
TMZ-sensitive	57	1.76 ± 0.15	
TMZ-resistant	29	2.63 ± 0.19	

### Down-regulated FOXD2-AS1 represses migration, proliferation, epithelial-mesenchymal transition (EMT) and invasion while stimulating apoptosis of glioma cells

**Figure 2:**
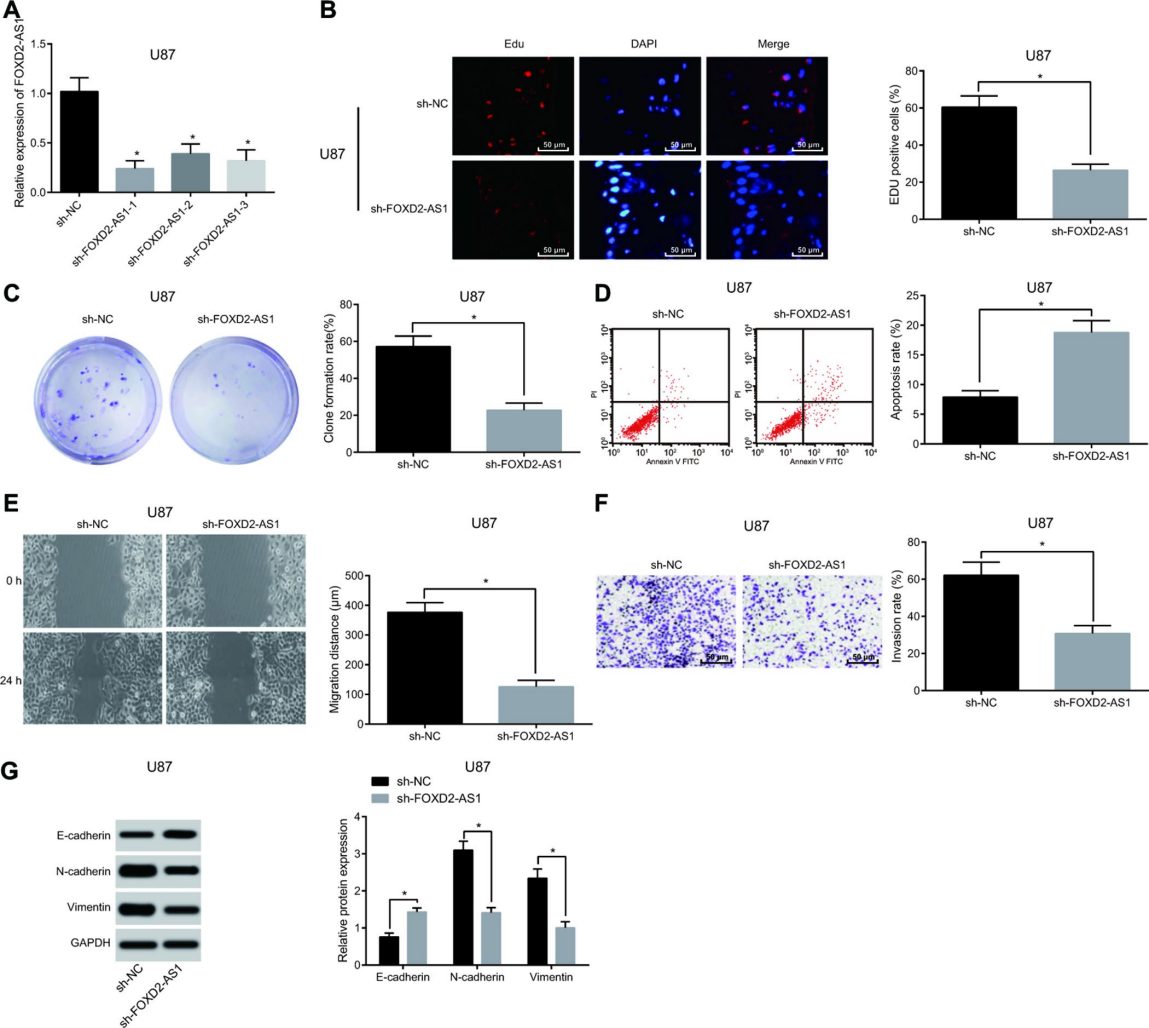
**Silencing of FOXD2-AS1 results in inhibition of the proliferation, migration, invasion and EMT of glioma U87 cells and promotion of their apoptosis (Data of U251 cells were shown in Supplementary Figure 1).** (**A**) The expression of FOXD2-AS1 in U87 cells were detected by RT-qPCR. (**B**) EdU assay was used to detect proliferation of U87 cells. (**C**) The ability of cell colony formation of U87 was detected by colony formation assay; (**D**) Flow cytometry was used to detect cell apoptosis of U87 cells in each group. (**E**) Cell migration ability of U87 cells was tested by scratch test; (**F**) Transwell assay was used to detect cell invasion of U87 cells in each group. (**G**) Western blot analysis was conducted to detect the expression of factors related to EMT in U87 cells. * *P* < 0.05 vs the sh-NC group; The data were all measurement data, represented by mean ± standard deviation. The comparison between the two groups was statistically analyzed by independent sample t test, and the experiment was repeated for three times.

FOXD2-AS1 expression in U87 and U251 cells was detected by RT-qPCR. Results (Figure 2A, Supplementary Figure 1A) suggested that in contrast with the sh-NC group, the expression of FOXD2-AS1 in the sh-FOXD2-AS1-1, sh-FOXD2-AS1-2 and sh-FOXD2-AS1-3 groups were significantly decreased, and the lowest of which was in the sh-FOXD2-AS1-1 group (*P* < 0.05). Therefore, sequence in the sh-FOXD2-AS1-1 group was selected to silence FOXD2-AS1 in subsequent experiments. For the effect of FOXD2-AS1 on the activity of glioma cells, EdU assay and colony formation assay were used to detect the cell proliferation and cell colony formation ability. The results (Figure 2B–2C, Supplementary Figure 1B, 1C) displayed that compared with the sh-NC group, the cell proliferation and colony formation rate in the sh-FOXD2-AS1 group were clearly reduced (both *P* < 0.05). Flow cytometry results (Figure 2D, Supplementary Figure 1D) showed that cell apoptosis was evidently increased in the sh-FOXD2-AS1 group (*P* < 0.05) when compared with the sh-NC group. The invasion and migration abilities of cells in each group were detected by scratch test and Transwell assay respectively, and the results indicated that (Figure 2E, 2F, Supplementary Figure 1E, 1F) in comparison with the sh-NC group, the invasion and migration of cells in the sh-FOXD2-AS1 group were distinctly lessened (both *P* < 0.05). Meanwhile, western blot analysis was employed to detect the expression of factors related to EMT, and the results indicated that (Figure 2G, Supplementary Figure 1G) in comparison with the sh-NC group, E-cadherin expression in the sh-FOXD2-AS1 group was overtly increased, while the expression of N-cadherin and Vimentin was significantly decreased (all *P* < 0.05), indicating that EMT was inhibited. The above results suggests that silencing FOXD2-AS1 contributes to the inhibition of the proliferation, colony formation, migration, invasion and EMT of glioma cells, and promotion of apoptosis.

### U87TR and U251TR have higher chemotherapy resistance to TMZ

**Figure 3:**
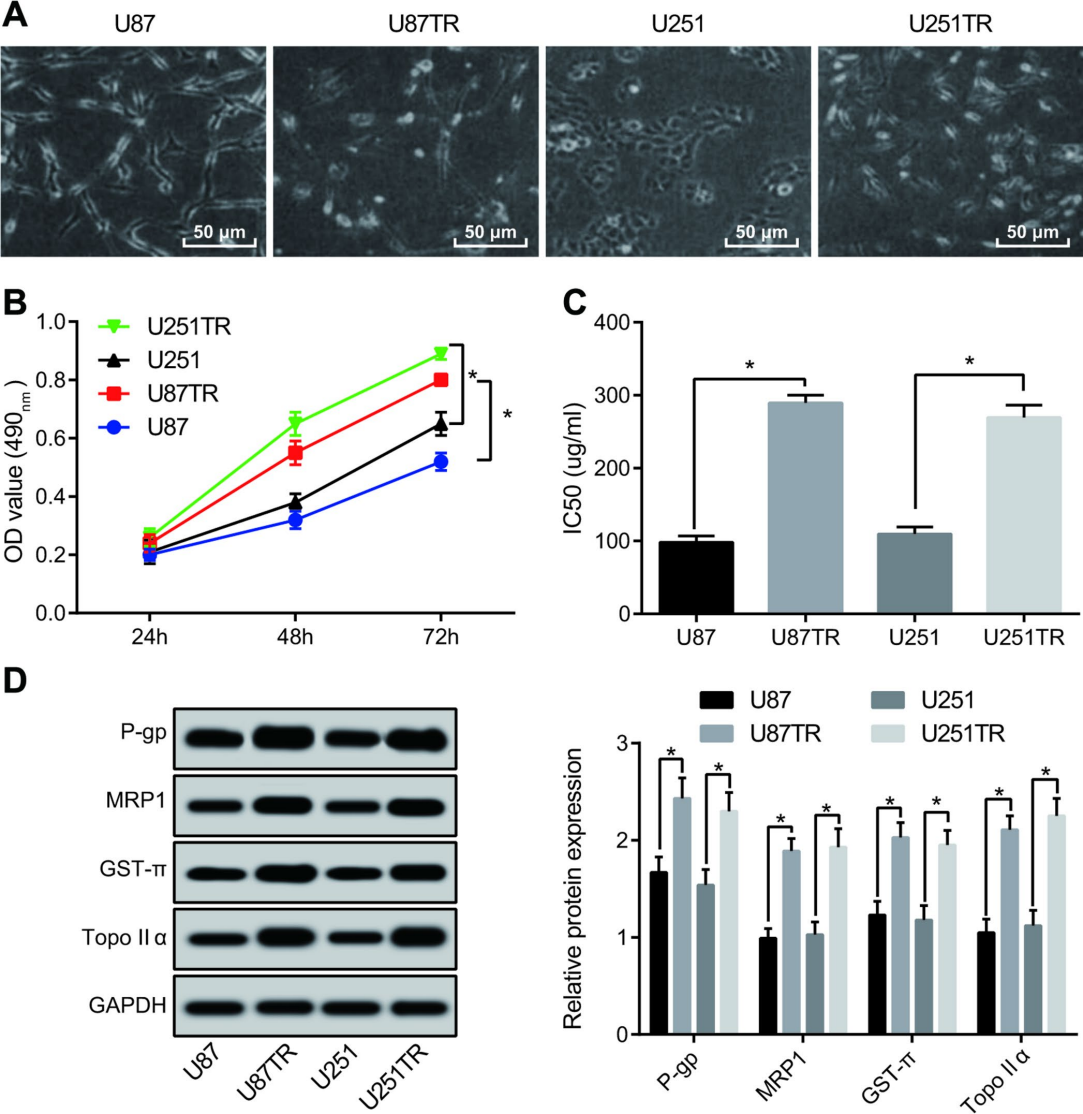
**TMZ resistant cell lines U87TR and U251TR show higher chemotherapeutic resistance than that of the parent cell lines U87 and U251.** (**A**) Morphological changes of drug-resistant cell lines U87TR and U251TR and parent cell lines U87 and U251 were observed by an optical microscope (× 200); (**B**) MTT assay was used to determine the cell proliferation capacity at the concentration of TMZ at 50 μg/mL; (**C**) Changes of parental cells and drug-resistant cells on IC50 of TMZ; (**D**) The expression of drug-resistant genes detected by western blot analysis; * *P* < 0.05 vs U87 cells or U251 cells; The data were all measurement data, represented by mean ± standard deviation. The comparison between the two groups was statistically analyzed by independent sample t test, and the experiment was repeated for three times.

The morphological changes of TZM-resistant cell lines U251TR and U87TR constructed by parental GBM cell lines U251 and U87 were shown in Figure 3A. In order to testify whether U87TR and U251TR were more resistant to TZM, MTT assay was carried out for detecting the cell proliferation capacity at the concentration of 50 μg/mL TZM. The results manifested that the proliferation capacity of U87TR and U251TR cells under the action of TZM was apparently stronger than that of parental cells (Figure 3B). Meanwhile, the results of the IC50 revealed that the IC50 of TZM in U87TR cells was 289.53 ± 10.54 μg/mL, 2.95 times of that in U87 cells. The IC50 of U251TR against TMZ was 269.57 ± 16.98 μg/mL, 2.46 times that of U251 cells (Figure 3C). Western blot analysis was adopted for detection of expression of drug resistance-associated genes simultaneously, and the results revealed that (Figure 3D) the expression of P-gp, MRP1, GST-π, and TopoIIα in drug resistant cell lines was increased to different extent than those in the parental cell lines. The above results suggests that the resistance of TMZ-resistant cell lines U87TR and U251TR to the chemotherapy of TMZ was distinctly higher than that of the parental cell lines.

### Silence of FOXD2-AS1 suppresses proliferation and drug resistance of drug-resistant glioma cells and promotes their apoptosis

**Figure 4:**
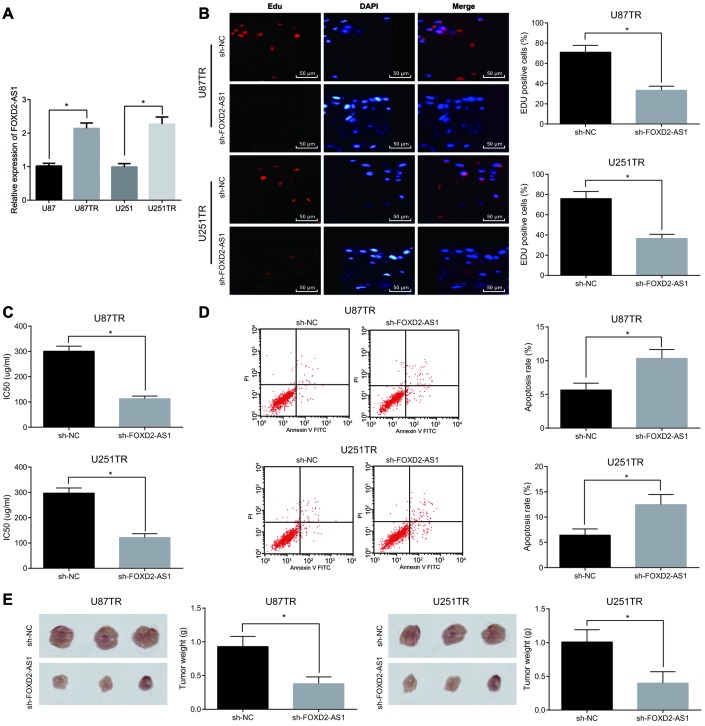
**Silencing of FOXD2-AS1 contributes to inhibition of proliferation and drug resistance of drug-resistant glioma cells and promotion of their apoptosis.** (**A**) The expression differences of FOXD2-AS1 in drug-resistant cell lines (U87TR, U251TR) and parental cell lines (U87, U251) were detected by RT-qPCR. (**B**) EdU assay was used to detect cell proliferation activity. (**C**) Changes of IC50 in drug-resistant cell lines after silencing FOXD2-AS1; (**D**) Cell apoptosis was detected by flow cytometry; (**E**) Tumor growth in nude mice was detected by tumor formation experiment; * *P* < 0.05 vs the sh-NC group; The data were all measurement data, represented by mean ± standard deviation. The comparison between the two groups was statistically analyzed by independent sample t test, and the experiment was repeated for three times.

The difference of FOXD2-AS1 expression in drug-resistant cell lines (U87TR, U251TR) and parental cell lines (U87, U251) was detected by RT-qPCR, and the results suggested that the expression of FOXD2-AS1 in drug-resistant cell lines was significantly higher than that in parental cell lines (*P* < 0.05) (Figure 4A). As to further investigate the effect of FOXD2-AS1 on the drug-resistant glioma cell lines, low expression of FOXD2-AS1 drug-resistant cell lines was constructed and EdU assay was employed to detect the cell proliferation in each group. The results indicated that in contrast with the sh-NC group, the cell proliferation in the sh-FOXD2-AS1 group was evidently reduced (*P* < 0.05) (Figure 4B). In the meantime, the results of IC50 showed that (Figure 4C) the IC50 of cells was significantly decreased after silencing FOXD2-AS1 (*P* < 0.05). Flow cytometry was used to detect cell apoptosis (Figure 4D), and the apoptosis rate was clearly increased after silencing FOXD2-AS1 (*P* < 0.05). Meanwhile, the tumor weight was tested after silencing FOXD2-AS1 *in vivo*, and the results indicated that tumor weight was apparently inhibited by decreasing FOXD2-AS1 (Figure 4E), and the same trend was found in the cell lines U87TR and U251TR. The above results reveals that silencing of FOXD2-AS1 suppresses proliferation, drug resistance and tumor growth of glioma drug resistant cells, and promotes their apoptosis.

### Overexpressed FOXD2-AS1 promotes the proliferation and drug resistance of drug-resistant cells of glioma and inhibits their apoptosis

**Figure 5:**
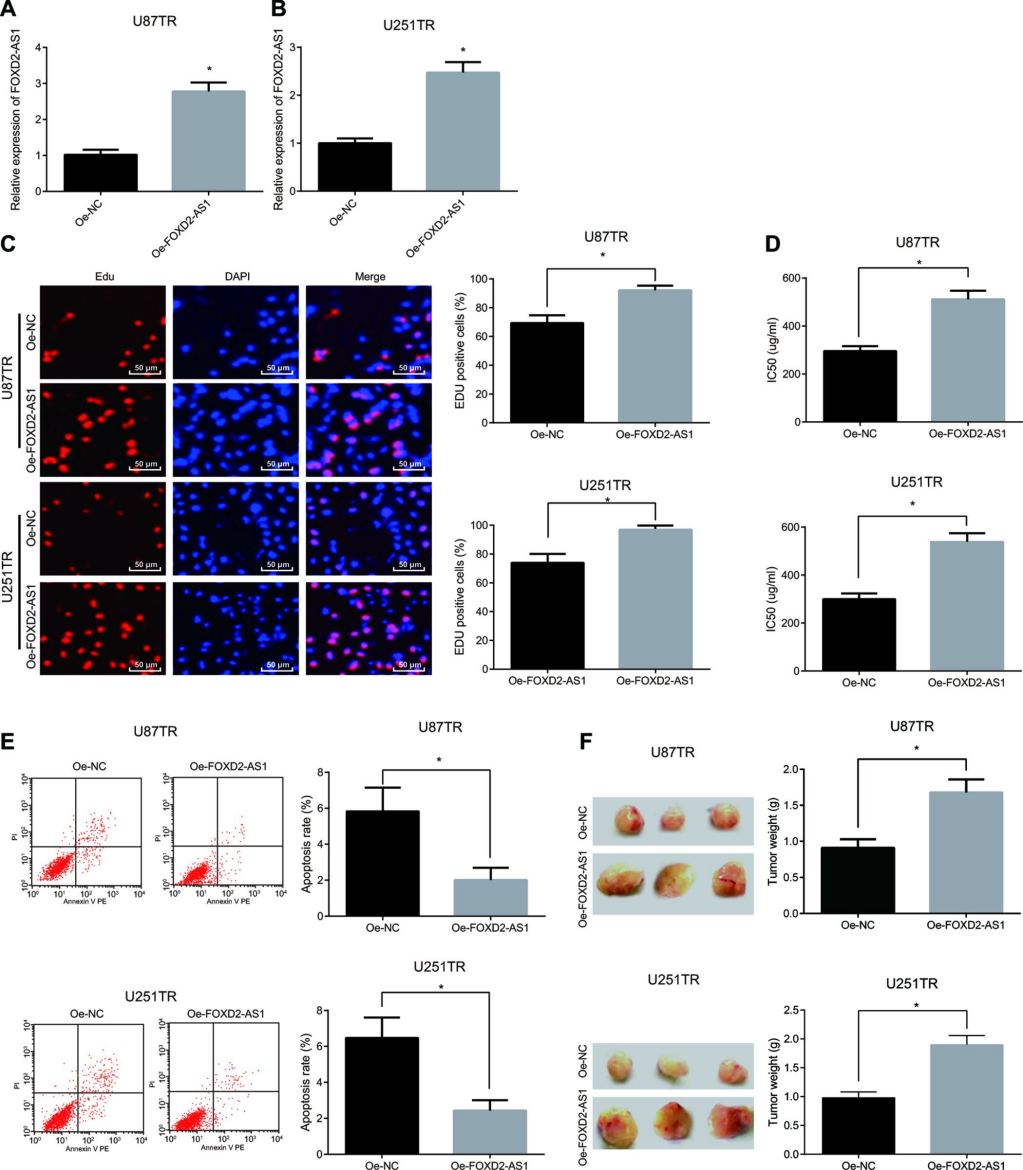
**Overexpression of FOXD2-AS1 accelerates the proliferation and drug resistance of drug-resistant cells in glioma and inhibits their apoptosis.** (**A**) The expression of FOXD2-AS1 in U87TR cell line was detected by RT-qPCR; (**B**) The expression of FOXD2-AS1 in U251TR cell line was examined by RT-qPCR; (**C**) The cell proliferation activity tested via EdU assay; (**D**) The changes of IC50 in drug-resistant cell lines after overexpression of FOXD2-AS1; (**E**) The apoptosis in U87TR and U251TR cell lines tested via flow cytometry; (**F**) The tumor growth tested via tumor xenograft in nude mice. * *P* < 0.05 vs the oe-NC group; The data in the figure were all measurement data, represented by mean ± standard deviation. The comparison between the two groups was statistically analyzed by independent sample t test, and the experiment was repeated for three times.

After overexpression of FOXD2-AS1, the expression of FOXD2-AS1 in drug-resistant cell lines (U87TR and U251TR) were detected by RT-qPCR. The results suggested that (Figure 5A–5B) in contrast with the oe-NC group, the FOXD2-AS1 expression elevated overtly in the oe-FOXD2-AS1 group (*P* < 0.05). In order to further discuss the effect of FOXD2-AS1 on the drug-resistant cell lines in glioma, the drug-resistant cell lines with up-regulated FOXD2-AS1 were established, and the proliferation of the cells was detected by the EdU assay. The results revealed that (Figure 5C) in contrast with the oe-NC group, the proliferation of the cells in the oe-FOXD2-AS1 group increased apparently (*P* < 0.05). At the same time, the IC50 results manifested that (Figure 5D) the IC50 of the cells after overexpressing FOXD2-AS1 was obviously elevated (*P* < 0.05). The results of flow cytometry demonstrated that (Figure 5E) the apoptosis rate was overtly declined after overexpressing FOXD2-AS1 (*P* < 0.05). In addition, the tumor weight was detected after the overexpression of FOXD2-AS1 *in vivo*, and the results showed that the up-regulated FOXD2-AS1 overtly increased tumor weight (Figure 5F), and U87TR and U251TR cell lines showed the same trend. The above results indicate that the overexpression of FOXD2-AS1 promotes the proliferation, drug resistance and tumor growth *in vivo* of drug-resistant cells in glioma, and inhibits their apoptosis.

### LncRNA FOXD2-AS1 acts as a competing endogenous RNA (ceRNA) of miR-98-5p

**Figure 6:**
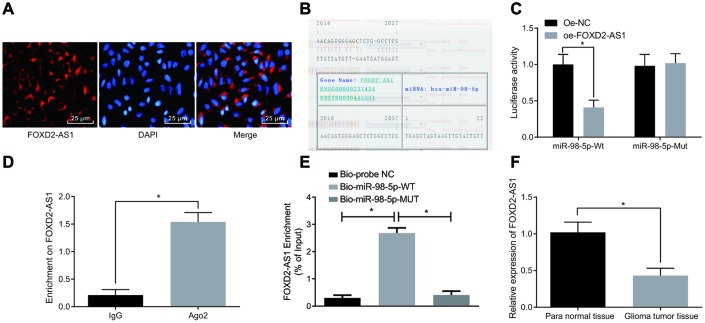
**LncRNA FOXD2-AS1 is regarded as a sponge of miR-98-5p.** (**A**) The subcellular localization of FOXD2-AS1 was verified by FISH assay. (**B**) The binding site of FOXD2-AS1 and miR-98-5p were predicted in the RNA22 website. (**C**) The binding of FOXD2-AS1 to miR-98-5p was verified by dual luciferase reporter gene assay, miR-98-5p-MUT was MUT plasmid of FOXD2-AS1 and miR-98-5p 3′UTR, and miR-98-5p-WT was WT plasmid of FOXD2-AS1 and miR-98-5p 3′UTR; (**D**) The binding of FOXD2-AS1 to Ago2 was detected by RIP assay. (**E**) RNA pull-down assay was used to detect the enrichment of FOXD2-AS1 by miR-98-5p. (**F**) Expression of miR-98-5p in glioma tumor tissues was detected by RT-qPCR, N = 86; * *P* < 0.05 vs the oe-NC group, IgG group, Bio-probe NC group or para normal tissues; The data were all measurement data, represented by mean ± standard deviations. The comparison between the two groups was statistically analyzed by independent sample t test, and one-way ANOVA was used in comparisons among multiple groups, and Tukey’s post-hoc test was performed after ANOVA. The experiment was repeated for three times.

The subcellular localization of lncRNAs is closely related to their biological function and potential molecular roles. First, we detected the subcellular localization of FOXD2-AS1 via using RNA-FISH and the results manifested that FOXD2-AS1 was indeed concentrated in the cytoplasm (Figure 6A), indicating that FOXD2-AS1 may function in the cytoplasm. It was found through RNA22 website (https://cm.jefferson.edu/rna22/Precomputed/) that FOXD2-AS1 could combine with miR-98-5p (Figure 6B). Dual luciferase reporter gene assay found that in comparison with the oe-NC group, luciferase activity of miR-98-5p-WT in the oe-FOXD2-AS1 group decreased significantly (*P* < 0.05); there was no distinct difference in luciferase activity of miR-98-5p-MUT (*P* > 0.05), suggesting that there may a specific binding site between miR-98-5p and FOXD2-AS1 (Figure 6C). The relationship between FOXD2-AS1 and Ago2 was detected by RIP assay, and the results indicated that compared with the IgG group, the specific adsorption level of FOXD2-AS1 toward Ago2 was obviously increased (*P* < 0.05; Figure 6D). The RNA pull-down assay indicated that FOXD2-AS1 could be used as a ceRNA to adsorb miR-98-5p. The results showed that by contrast with the Bio-probe NC group, the enrichment of FOXD2-AS1 was evidently increased in the Bio-miR-98-5p-WT group (*P* < 0.05), while there was no apparent difference in the enrichment of FOXD2-AS1 in the Bio-miR-98-5p-MUT group (*P* > 0.05; Figure 6E). Meanwhile, miR-98-5p expression in glioma tumor tissues was detected by RT-qPCR, and the results manifested that miR-98-5p expression in glioma tumor tissues was clearly lower than that in the para normal tissues (*P* < 0.05; Figure 6F). The above results suggest that lncRNA FOXD2-AS1 acts as a ceRNA to adsorb miR-98-5p, thus decreasing the expression of miR-98-5p.

### Overexpression of FOXD2-AS1 suppresses expression of miR-98-5p, thereby promoting the expression of CPEB4

**Figure 7:**
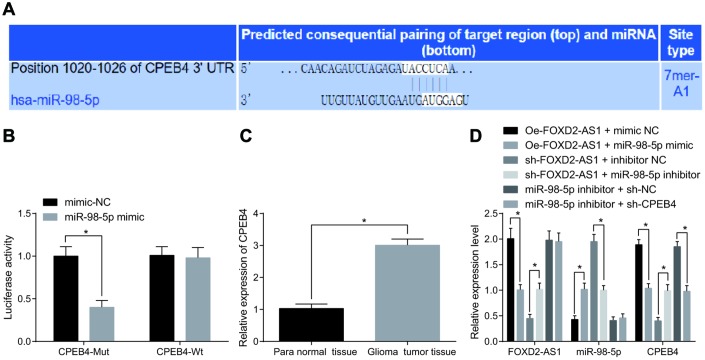
**FOXD2-AS1 acts as a ceRNA to adsorb miR-98-5p, thereby decreasing the expression of CPEB4.** (**A**) The target relationship between miR-98-5p and CPEB4 was predicted by Targetscan website. (**B**) The targeting relationship between miR-98-5p and CPEB4 was verified through dual luciferase reporter gene assay, CPEB4-MUT was MUT plasmid of miR-98-5p and CPEB4 3′UTR, and CPEB4-WT was WT plasmid of miR-98-5p and CPEB4 3′UTR; (**C**) CPEB4 expression in glioma tumor tissues was detected by RT-qPCR, N = 86; (**D**) The expression levels of FOXD2-AS1, miR-98-5p and CPEB4 in cells after transfection were detected by RT-qPCR. * *P* < 0.05 vs the mimic-NC group, para normal tissues, the oe-FOXD2-AS1 + mimic NC group, the sh-FOXD2-AS1 + inhibitor NC group or the miR-98-5p inhibitor + sh-NC group; The data were all measurement data, represented by mean ± standard deviation. The comparison between the two groups was statistically analyzed by independent sample t test, and the experiment was repeated for three times.

In Targetscan website (http://www.targetscan.org/vert_71/), the target genes of miR-98-5p was predicted, the finding revealed that there was binding sites between miR-98-5p and CPEB4 (Figure 7A). At the same time, the detection of dual luciferase reporter gene assay indicated that (Figure 7B) there was specific binding site between miR-98-5p and CPEB4, and CPEB4 was the target gene of miR-98-5p. It is speculated that miR-98-5p expression was inhibited after being adsorbed by FOXD2-AS1, thereby upregulating the expression of CPEB4. The expression of CPEB4 was detected in glioma tumor tissues and the para normal tissues, and the results suggested that CPEB4 expression in glioma tumor tissues was evidently higher than that in the para normal tissues (*P* < 0.05; Figure 7C). Meanwhile, RT-qPCR was performed on the cells after transfection, and the expression levels of FOXD2-AS1, miR-98-5p and CPEB4 in each group were detected. The results showed (Figure 7D) compared with the oe-FOXD2-AS1 + mimic NC group, the expression of FOXD2-AS1 and CPEB4 were inhibited in the oe-FOXD2-AS1 + miR-98-5p mimic group (both *P* < 0.05). In comparison with the sh-FOXD2-AS1 + inhibitor NC group, the expression of FOXD2-AS1 and CPEB4 was promoted in the sh-FOXD2-AS1 + miR-98-5p inhibitor group (both *P* < 0.05). Compared with the miR-98-5p inhibitor + sh-NC group, no change in the expression of FOXD2-AS1 and miR-98-5p was found in the miR-98-5p inhibitor + sh-CPEB4 group (both *P* > 0.05), while through which the expression of CPEB4 was decreased (*P* < 0.05). The above results were enunciated that FOXD2-AS1 could be used as a ceRNA to adsorb miR-98-5p and inhibit the expression of miR-98-5p, thus strengthening the expression of CPEB4. However, the expression of CPEB4 was inhibited by decline of FOXD2-AS1 or up-regulation of miR-98-5p.

## DISCUSSION

Gliomas are the most frequent primary brain tumors, and the malignant tumor in the central nervous system [10, 16]. Several reports have shown that miRNAs and lncRNAs were closely linked to glioma. For example, miRNAs have been demonstrated to exerts functions in invasion and migration of glioma cells [14]. Besides, a study has found that there are a number of lncRNAs are involved in the initiation of glioma [20]. Based on this, this purpose of study was to investigate the functions of lncRNA FOXD2-AS1 in glioma and its mechanism on the biological features of glioma cells and the drug resistance of TMZ. Results in this study demonstrated that silencing lncRNA FOXD2-AS1 inhibited migration, invasion, proliferation and drug resistance of drug-resistant glioma cells and promoted their apoptosis via enhancing the expression of miR-98-5p or decreasing CPEB4.

The major finding of this study showed that highly expressed FOXD2-AS1 was found in human glioma tissues and cells. Other studies also confirmed that FOXD2-AS1 overexpression was existed in glioma cell line [9, 10]. Our study also demonstrated that down-regulated FOXD2-AS1 repressed proliferation, colony formation, migration, invasion and EMT (significantly increased E-cadherin expression, clearly declined N-cadherin and Vimentin expression) while stimulating apoptosis of glioma cells. Additionally, overexpressed FOXD2-AS1 apparently promoted the proliferation and drug resistance of drug-resistant cells of glioma and inhibited their apoptosis. In accordance with the present results, a previous study has demonstrated that knockdown of FOXD2-AS1 distinctly decreased glioma cell EMT, invasion, proliferation and migration, and promoted apoptosis [9]. As demonstrated in a previous study, the similar results were obtained, which showing that knockdown of FOXD2-AS1 promoted the epithelial marker E-cadherin protein expression and declined Snail protein and N-cadherin expression [21]. In addition, another study has revealed that FOXD2-AS1 downregulation inhibited the migration and proliferation of glioma cells [22]. What’s more, from this study, we also found that silence of FOXD2-AS1 obvious inhibited proliferation and drug resistance of drug-resistant glioma cells and promoted their apoptosis. Evidence has shown that the gemcitabine-resistance of bladder cancer was accelerated by lncRNA FOXD2-AS1 through sponging miR-143 [23].

The present study indicated that miR-98-5p expression in glioma tumor tissues was clearly lower than that in para normal tissues. As previously reported, there was downregulated miR-98 in glioma cell lines and glioma tissues in contrast with their normal counterparts [13, 14]. Another interesting finding was that there was significantly higher CPEB4 expression in glioma tumor tissues than that in para normal tissues. Evidence has also demonstrated that CPEB4 is highly expressed in human glioma [18, 19]. Another important finding was that FOXD2-AS1 could be used as a ceRNA to adsorb miR-98-5p for inhibiting its expression, thereby promoting the expression of CPEB4. Additionally, this study revealed that FOXD2-AS1 contributed to the inhibition of miR-98-5p. There were some studies indicating the relationship between FOXD2-AS1 and miRNAs. This also accorded with the earlier observations, which displayed that FOXD2-AS1 was regarded as a ceRNA through sponging miR-7-5p [24]. Meanwhile, lncRNA FOXD2-AS1 was verified to act as a sponge of miR-185-5p [22]. Moreover, it has been also demonstrated that glioma tumorigenesis and malignancy were facilitated by upregulated FOXD2-AS1 through downregulating miR-185-5p and increasing CCND2 expression [10]. Except that, the results of this current study indicated that CPEB4 expression was inhibited by miR-98-5p, and CPEB4 was determined to be a target gene of miR-98-5p. There were some studies showing the relationship between miRNAs and CPEB4. For example, a study has demonstrated that CPEB4 is a novel target of miR-203, and the anti-tumor influence of miR-203 was reversed via overexpression of CPEB4 in SW480 cells [25]. In the meantime, Sun et al. have found that the expression of CPEB4 was significantly inhibited by enforced expression of miR-29c-5p [26].

In summary, our study concluded that silencing FOXD2-AS1 upregulated miR-98-5p and decreased CPEB4, thus obviously inhibiting the proliferation, migration, invasion and drug resistance of drug-resistant glioma cells and promoting their apoptosis. Investigation of role of FOXD2-AS1 in drug resistance of glioma yielded a better understanding of their inner mechanisms and may have potential therapeutic implications in the treatment of glioma. In the future, the results of the study can be further verified by enlarging the sample size. Meanwhile, the key mechanism for how FOXD2-AS1 works in glioma needs further verification.

## MATERIALS AND METHODS

### Ethics statement

The study was approved by the Institutional Review Board of Xi’ an Jiaotong University Health Science Center and followed the tenets of *the Declaration of Helsinki*. Written informed consent was acquired from each participant. This experiment was permitted by the International Convention on Laboratory Animal Ethics of Xi’an Jiaotong University Health Science Center.

### Study subjects

From January 2017 to January 2018, a total of 86 glioma tumor tissues and its para normal tissues were collected in Jiaotong University Health Science Center. According to the WHO staging criteria [27], 62 cases were in I + II stage and 24 cases were in III + IV stage. The 86 patients were assigned into TMZ-sensitive group (57 cases), and TMZ-resistant group (29 cases) according to computed tomography and response evaluation criteria in solid tumors (RECIST) [28]. None of the patients accepted chemoradiotherapy before the operation. The collected tissues were immediately frozen and preserved in liquid nitrogen for further use.

### Cell culture

Human normal glial brain cell lines HEB and human glioma cell lines (U87, U251, LN229, A172) were purchased from Shanghai Institute of Cells of Chinese Academy of Sciences (Shanghai, China). The cells were incubated in Dulbecco’s modified Eagle’s Medium (DMEM) supplemented with 10% fetal bovine serum (FBS) and penicillin-streptomycin (Gibco by Life technologies, Grand Island, NY, USA) and cultured in an incubator at 37 °C with 5% CO_2_. Cells were detached with 0.25% trypsin and passaged in 1:3 and then seeded into 6-well plates (3 × 10^5^ cells per well). When reaching 70% to 80% confluence, cells in logarithmic growth phase were taken for subsequent experiments.

### Cell grouping and transfection

The logarithmic growth phase cells (2 × 10^5^ cells per well) were seeded into a 6-well cell culture plate and transfected when the cells adhered to the wall and the cell confluence reached 30%-60%. According to the instructions of Lipofectamine 2000 kit (11668-027, Invitrogen, Carlsbad, CA, USA), 250 μL serum-free DMEM culture medium was diluted with each transfection sequence (Shanghai GenePharma Co.,Ltd., Shanghai, China, the final concentration was 50 nM in cells), and another 250 μL serum-free DMEM culture medium was diluted with 5 μL lipofectamine 2000. The above two were mixed and incubated at room temperature for 20 minutes before adding them into the cell culture well. After being incubated for 6 hours at 37 °C, with 5% CO_2_ and saturated humidity, the medium containing transfection solution in wells was replaced by DMEM medium containing 10% FBS for further culture. The cells were classified into sh-negative control (NC) (transfection of silenced FOXD2-AS1 NC plasmid), sh-FOXD2-AS1 (transfection of silenced FOXD2-AS1 plasmid), overexpression (oe)-NC (transfection of FOXD2-AS1 overexpressing NC plasmid), oe-FOXD2-AS1 (transfection of FOXD2-AS1 overexpressing plasmid), mimic-NC (transfection of miR-98-5p overexpressing NC plasmid), miR-98-5p mimic (transfection of miR-98-5p overexpressing plasmid), inhibitor-NC (transfection of miR-98-5p inhibitor NC plasmid), miR-98-5p inhibitor (transfection of miR-98-5p inhibitor plasmid), oe-FOXD2-AS1 + mimic NC group (transfection of oe-FOXD2-AS1 and miR-98-5p mimic NC plasmid), oe-FOXD2-AS1 + miR-98-5p mimic group (transfection of oe-FOXD2-AS1 and miR-98-5p mimic plasmid), sh-FOXD2-AS1 + inhibitor NC group (transfection of sh-FOXD2-AS1 and miR-98-5p inhibitor NC plasmid), sh-FOXD2-AS1 + miR-98-5p inhibitor group (transfection of sh-FOXD2-AS1 and miR-98-5p inhibitor plasmid), miR-98-5p inhibitor + sh-NC group (transfection of miR-98-5p inhibitor and sh-CPEB4 NC plasmid) and miR-98-5p inhibitor + sh-CPEB4 group (transfection of miR-98-5p inhibitor and sh-CPEB4 plasmid).

### Drug resistance model of human glioma cells induced by high dose shock

U87 and U251 cells in logarithmic growth phase were seeded in a 250 mL culture flask. When cells reached 70%-80% confluence, the cells were added with TMZ until the final concentration was 5 μg/mL. After 2 hours of treatment, the drug-containing medium was discarded and the living cells were then seeded in a new culture bottle. When the cells reached 70%-80% confluence, the above steps were repeated until the cell death rate was less than 5% at the mass concentration of TMZ of 0.4-0.5 μg/mL. The drug-resistant cell lines were named U87TR and U251TR, respectively.

### 5-ethynyl-2′-deoxyuridine (EdU) assay

Glioma cells transfected with plasmids and interfering RNA were inoculated into 96-well plates with 4 × 10^3^ cells per well. The proliferation of cells was detected by EdU kit (Ribobio, Guangzhou, China) when reaching 80% confluence. The original culture medium was renewed by 100 μL 50 μm EdU medium (EdU solution diluted by 1000:1 in cell culture medium) and incubated for 2 hours. The former medium was discarded, and then washed by phosphate buffered saline (PBS) twice, 5 min/time. The cells were incubated at fixed room temperature for 5 minutes with 50 μL 2 mg/mL glycine after 30-min fixation with 50 μL 4% paraformaldehyde and 5-min PBS washing. Subsequently, the cells were incubated with 100 μL 0.5% TritonX-100 for 10 minutes per well. After incubating at room temperature for 30 minutes in dark, cells were stained with 100 μL 1× Apollo dyeing solution and decolorized with methanol. After the nucleus were stained with 4′,6-diamidino-2-phenylindole (DAPI), a laser confocal microscopy (Leica, Heidelberg, Germany) was used for observation.

### 3-[4,5-dimethylthiazol-2-yl]-2,5-diphenyl tetrazolium bromide (MTT) assay

Cells were seeded into 96-well plates at 1 × 10^4^ cell/well and incubated overnight at 37 °C with 5% CO_2_. In the medium consisting of 10% FBS, cells were treated with TMZ (KeyGen Biotech Co., Ltd., Nanjing, China), a chemotherapeutic drug of 0, 4, 20, 100, 500, or 2500 μg/mL, for 24 hours respectively. The inhibited concentration of half of the cells (IC50) of TMZ was also detected. Then, 10 μL MTT solution (0.5 mg/mL) was added to each well and incubated for 4 hours. Then, The reaction was terminated by adding 200 μL dimethyl sulfoxide (DMSO) and incubated at 37 °C for 15 minutes after removing the supernatant. The optical density (OD) value at 490 nm was measured by a microplate reader (Bio-Rad, Hercules, CA, USA).

### Colony formation assay

The transfected cells were seeded into 6-well plates at 400 cells per well for colony formation assay. After 7-14 days of culture, the culture was terminated when the colonies were visible. The culture medium was absorbed before PBS cleaning, then fixed with methanol for 30 minutes, and stained with 0.1% crystal violet staining solution. The colony imaging was captured for counting the colony formation rate.

### Flow cytometry

Apoptotic cells after transfection were detected by Annexin V-fluorescein isothiocyanate (FITC)/propidium iodide (PI) double staining. Glioma cells were inoculated into 6-well plates with 2 × 10^5^ cells/well. After being transfected for 72 hours, the culture medium was removed and then washed with pre-cooled PBS at 4°C once, lastly detached with trypsin. The cells were collected in a 15 mL centrifugal tube. After centrifugation (800 g), the supernatant was removed and the cells were washed and precipitated twice with PBS. According to the instructions of Annexin V-FITC Apoptosis Detection Kit Ι (BD Company, Lake Franklin, New Jersey, USA), the cells were suspended in 500 μL binding buffer, and mixed with 5μL FITC and 5 μL PI in darkness. Finally, the cells were incubated for 15 minutes and their apoptosis was detected by flow cytometry. The judgment criteria of results were as follows: with Annexin V as the horizontal axis and PI as the vertical axis, (Annexin V-FITC)-/PI+ was regarded as the upper left quadrant, where the cells were necrotic. There might be also a small number of late apoptotic cells, and even mechanically damaged cells. (Annexin V-FITC)+/PI+ was considered as the upper right quadrant, where the cells were lately apoptotic cells. In the lower right quadrant (Annexin V-FITC)+/PI-, the cells were early apoptotic cells. In the lower left quadrant (Annexin V-FITC)-/PI-, the cells were living cells. Apoptosis rate = [(early apoptotic cells + late apoptotic cells)/total number of cells] × 100%.

### Scratch test

After 48 hours of transfection, glioma cells were cultured in 5 × 10^5^ cells per well to 6-well plates. After the cells were completely adherent to the wall, glioma cells were scratched with a 2 mm cell scraper in the middle of each well and then cultured for 24 hours. Photographs were taken at 0 h and 24 h after scratching, and the scratch distance was computed with Image-Proplus6.0. The experiments were repeated three times at least.

### Transwell assay

After being transfected for 48 hours, glioma cells were detached in serum-free medium after starvation for 24 hours. Then the cells were suspended in serum-free Opti-MEMI medium (Invitrogen, Carlsbad, CA, USA) containing 10 g/L bovine serum albumin (BSA, Sigma-Aldrich, St Louis, MO, USA), and the cell density was adjusted to 3 × 10^4^ cells/mL. In the experiment, 8 μm Transwell chamber (Corning, NY, USA) in the 24-well plate was used with three chambers in each group, with 100 μL cell suspension in each chamber. The lower chamber was supplemented with 600 μL 10% DMEM medium and incubated at 37°C with 5% CO_2_. In the migration test, the chamber was fixed with 4% polyformaldehyde for 30 minutes, then treated in 0.2% Triton X-100 (Sigma-Aldrich, St Louis, MO, USA) solution for 15 minutes and stained with 0.05% gentian violet staining solution for 5 minutes. In the invasive experiment, 50 μL Matrigel (Sigma-Aldrich, St Louis, MO, USA) was coated in the cells before the experiment, and stained 48 hours later as mentioned above. The number of stained cells was counted under an inverted microscope for calculating the invasive rate.

### Dual luciferase reporter gene assay

The binding sites of FOXD2-AS1 and miR-98-5p were predicted through RNA22 website (https://cm.jefferson.edu/rna22/Precomputed/). The segments of miR-98-5p 3′untranslated region (UTR) binding sites were amplified by polymerase chain reaction (PCR). The synthetic target segments were cloned into pmiR-RB-REPORT^TM^ plasmid (RiboBio Co. Ltd., Guangzhou, China). Finally, the miR-98-5p 3′UTR wild-type (WT) plasmid (miR-98-5p-WT) was constructed. On the basis of this plasmid, the WT vector was used as the template for point mutation, and finally, the miR-98-5p 3′UTR mutant type (MUT) plasmid (miR-98-5p-MUT) was obtained. The 293T cells in logarithmic growth phase were seeded into the 96-well plate. When the cell confluence was about 70%, the vector of miR-98-5p-MUT and miR-98-5p-WT were co-transferred into 293T cells with oe-NC or oe-FOXD2-AS1 respectively via using Lipofectamine 2000. The cells were collected and lysed after transfection for 48 h, and centrifuged for 3-5 minutes. The supernatant was taken, and luciferase detection kit (RG005, Shanghai Beyotime Biotechnology Co., Ltd., Shanghai, China) was used respectively to determinate the relative lights units (RLU). The firefly luciferase to renilla luciferase ratios were determined and expressed as relative luciferase activity with firefly luciferase as the loading control. The experiment was repeated three times.

Bioinformatics software (http://www.targetscan.org/vert_72/) was utilized to predict the binding sites of miR-98-5p and CPEB4 3′UTR. CPEB4 3′UTR binding site fragment was amplified by PCR, and the amplified target gene fragment was cloned into pmiR-RB-REPORT^TM^ vector (RiboBio Co. Ltd., Guangzhou, China) for the construction of CPEB4 3′UTR WT plasmid (CPEB4-WT). Based on the plasmid, with WT vector as the template, two sequences including mutated sequences were amplified respectively. And then the amplified mutant sequences were cloned into pmiR-RB-REPORT^TM^ vector (RiboBio Co. Ltd., Guangzhou, China) for obtaining CPEB4 3′UTR MUT plasmid (CPEB4-MUT). The 293T cells in logarithmic growth phase were seeded into 96-well plates, and when the cell confluence reached about 70%, Lipofectamine 2000 was used to transfect CPEB4-MUT and CPEB4-WT vectors with mimic-NC or miR-98-5p mimic respectively to 293T cells. After 48-h transfection, the cells were collected and lysed, centrifuged for 3-5 minutes, and the supernatant was taken. The RLU was determined by luciferase detection kit (RG005, Shanghai Beyotime Biotechnology Co., Ltd., Shanghai, China). The firefly luciferase to renilla luciferase ratios were determined and expressed as relative luciferase activity with the firefly luciferase as the internal reference. The experiment was repeated three times.

### Fluorescence in situ hybridization (FISH)

Subcellular localization of FOXD2-AS1 was identified by FISH technique. According to the instructions of Ribo^TM^ lncRNA FISH Probe Mix (Red) (RiboBio Co., Ltd., Guangzhou, China), the specific method was as follows: Firstly, after the slides were placed in the 24-well culture plate, cells were taken and inoculated at 6 × 10^4^ cells/well for reaching 80% cell confluence. Slides were removed before PBS cleaning, and then 1 mL of 4% paraformaldehyde was added at fixed room temperature. After the reagent processing of proteinase K, glycine and acylation reagent, 250 μL pre-hybrid liquid was joined with 1-h incubation at 42°C. Secondly, 250 μL hybrid liquid of lncRNA FOXD2-AS1 (300 ng/mL) with probe was added and incubated overnight at 42°C after removing the hybrid liquid. After 3 times’ phosphate-buffered saline with Tween (PBST) rinsing, DAPI (ab104139, 1:100, Abcam, Shanghai, China) dye solution diluted with PBST was added for staining the nucleus. Then the cells were supplemented to the 24-well culture plate for 5-min dyeing, sealed with an anti-fluorescence quenching agent and observed and photographed under a fluorescence microscope (Olympus, Tokyo, Japan).

### RNA immunoprecipitation (RIP) assay

The binding of FOXD2-AS1 to Ago2 protein was detected by RIP kit (Millipore, Schwalbach, Germany). Glioma cells were washed with precooled PBS and the supernatant was discarded. The cells were cracked with equal volume of radioimmunoprecipitation assay (RIPA) lysate, and supernatant was taken through 10-min centrifugation at 14000 rpm/4°C. Later, co-precipitation was carried out with antibody incubation. The specific steps were as follows: 50 μL magnetic beads were taken from each co-precipitation reaction system and re-rotated into 100 μL RIP Wash Buffer. According to the experimental group, 5 μg antibody was added with incubation for binding. After cleaning and suspension in 900 μL RIP Wash Buffer in magnetic beads-antibody complex, 100 μL cells extracts were added and incubated at 4°C for the night. The sample was placed on a magnetic pedestal to collect the bead-protein complex. RNA was extracted for subsequent reverse transcription polymerase chain reaction (RT-qPCR) after the samples were detached by protease K. The antibodies used for RIP were rabbit anti-Ago2 (ab186733, 1:50, Abcam, Shanghai, China) and rabbit anti-IgG (ab109489, 1:100, Abcam, Shanghai, China) as a NC. The experiment was repeated three times.

### RNA pull-down assay

WT-bio-miR-98-5p and MUT-bio-miR-98-5p (Wuhan GeneCreate Biological Engineering Co., Ltd., Wuhan, China) labeled with 50 nM biotin were transfected into the cells. After 48 hours, the cells were collected, washed with PBS and incubated in specific lysis buffer (Ambion, Austin, Texas, USA) for 10 min. The lysate was incubated with M-280 streptavidin magnetic beads (S3762, Sigma-Aldrich, St Louis, MO, USA) precoated by RNase-free BSA and yeast tRNA (TRNABAK-RO, Sigma-Aldrich, St Louis, MO, USA) at 4°C for the night. The cells were rinsed with precooled lysis buffer twice, low-salt buffer for three times and high-salt buffer for one time. The bound RNA was purified by Trizol, and then FOXD2-AS1 enrichment was tested by RT-qPCR. The experiment was repeated three times.

### RT-qPCR

Total RNA was extracted from cells and tissues by Trizol (Takara, Dalian, China) method for detecting the concentration and purity of RNA. According to the instructions of the reverse transcription kit (K1621, Fermentas, Maryland, NY, USA), the RNA was reversely transcripted into cDNA. The primer sequences of FOXD2-AS1, miR-98-5p, and CPEB4 were synthesized by Shanghai Genechem Co., Ltd. (Shanghai, China (Table 2). The mRNA expression of each gene was detected by the fluorescence quantitative PCR kit (Takara, Dalian, China) by using a RT-qPCR instrument (ABI 7500, ABI, Foster City, CA, USA). The internal reference of miR-98-5p was U6 and the internal reference of FOXD2-AS1 and CPEB4 was glyceraldehyde phosphate dehydrogenase (GAPDH). The 2^-ΔΔCt^ method was conducted for the calculation of the relative gene expression, and each experiment was repeated three times.

**Table 2:** Primer sequence.

**Gene**	**Sequence (5′-3′)**
FOXD2-AS1	Forward: 5′-CACTGAGGGACAGCCAAGA-3′
	Reverse: 5′-GGCGGCGTGTAATTGGTA-3′
miR-98-5p	Forward: 5′-GGAAAATCGCCATAGCCAGG-3′
	Reverse: 5′-AGATCAGGGTGGCCCCATTT-3′
CPEB4	Forward: 5′-ACAGTGACTTTGTGAGATGGATGG-3′
	Reverse: 5′-TTATCATCGCAAGCTCCACA-3′
U6	Forward: 5′-CTCGCTTCGGCAGCACA-3′
	Reverse: 5′-AACGCTTCACGAATTTGCGT-3′
GAPDH	Forward: 5′-TCCCATCACCATCTTCCA-3′
	Reverse: 5′-CATCACGCCACAGTTTTCC-3′

Note: miR-98-5p, microRNA-98-5p; GAPDH, glyceraldehyde phosphate dehydrogenase.

### Western blot analysis

The tissues were added with 100 μL RIPA lysate (R0020, Beijing Solarbio Technology Co., Ltd., Beijing, China) and placed in a centrifuge tube. The tissues were homogenized at 3000 r/min until being fully cracked, and placed on the ice for 30 minutes at 4°C. Then the protein concentration was determined according to the instructions of bicinchoninic acid (BCA) kit (AR0146, Boster Biological Technology Co., Ltd., Wuhan, China), and the sample concentration was adjusted to 3 μg/L. The sample buffer was added into the extracted protein and the liquid was boiled at 95°C for 10 minutes.

Proteins were separated by 10% polyacrylamide gel electrophoresis and transferred to polyvinylidene difluoride (PVDF) membrane (P2438, Sigma-Aldrich, St Louis, MO, USA) and sealed with 5% BSA (10–116, Beijing Zhongsheng Likang technology Co., Ltd., Beijing, China) at room temperature for 1 h. Rabbit anti-E-cadherin (ab1416, 1:100), N-cadherin (ab18203, 1:100), Vimentin (ab193555, 1:100), P-glycoprotein (P-gp) (ab103477, 1:500), multidrug resistance-associated protein 1 (MRP1) (ab233383, 1:1000), Glutathione S-transferase (GST)-π (ab19256, 1: 1000), and topoisomerase IIα (TopoIIα) (ab52934, 1: 10000) were added and incubated at 4°C overnight, followed by the addition of the corresponding goat anti-rabbit secondary antibody (ab6721, 1:2000, Abcam, Cambridge, USA). Then the solution was incubated at room temperatu re for 1 h. The membrane was developed through chemiluminescence reagent with the internal reference of GADPH (ab181602, 1:10,000, Abcam, Cambridge, USA) by using Gel Doc EZ imager (Bio-rad, California, USA). Eventually, the gray value of the target band was analyzed by the Image J software.

### Tumor xenograft in nude mice

BALB/c nude mice (J004, BetterBiotechnologyCo., Ltd., Nanjing, China), aging 3-4 weeks, weighting 14 to 18 g, were fed under specific pathogen free condition. For example, all fodders were under the condition of the sterilization processing, and all experimental animals were under 18 to 22°C of feeding temperature and 50-60% of humidity. Nude mice were randomly divided into sh-NC group (injected with silenced FOXD2-AS1 NC sequence) and sh-FOXD2-AS1 group (injected with silenced FOXD2-AS1 sequence). Drug-resistant cell lines were detached with trypsin and made into cell suspension and the cell density was adjusted to 1 × 10^5^ cells/mL. Local skin of nude mice was disinfected. A total of 0.5 mL of cell suspension was subcutaneously injected into the thigh root of nude mice. The general situation and the local situation at the inoculation site were observed. Tumor volume was measured every other week with a vernier caliper, and all nude mice were euthanized at 6 weeks after injection. In the end, the tumor was obtained to observe the tumor gross specimen, and the tumor weight was measured for the plotted growth curve, and the tumor weight of each group was compared.

### Statistical analysis

Data were analyzed by using the SPSS21.0 software package (IBM SPSS Statistics, Chicago, IL, USA). All data were conformed to the normal distribution and homogeneity of variance. The measurement data were expressed as mean ± standard deviation. Independent sample t test was employed for the comparison between the two groups, and one-way analysis of variance (ANOVA) was used for comparisons among multiple groups. Tukey’s post hoc test was performed after ANOVA, and *P* < 0.05 was deemed statistically significant.

### Ethical statement

The experiment was approved by Jiaotong University Health Science Center.

## Supplementary Material

Supplementary Figure 1
