# Supplementary material for: Silencing lncRNA FOXD2-AS1 inhibits proliferation, migration, invasion and drug resistance of drug-resistant glioma cells and promotes their apoptosis via microRNA-98-5p/CPEB4 axis
Source: Aging (Albany NY). 2019 Nov 26;11(22):10266–83. doi: 10.18632/aging.102455 (PMC6914387; doi:10.18632/aging.102455)
Supplement: Supplementary Figure 1 [file aging-11-102455-s001..pdf]

SUPPLEMENTARY FIGURE

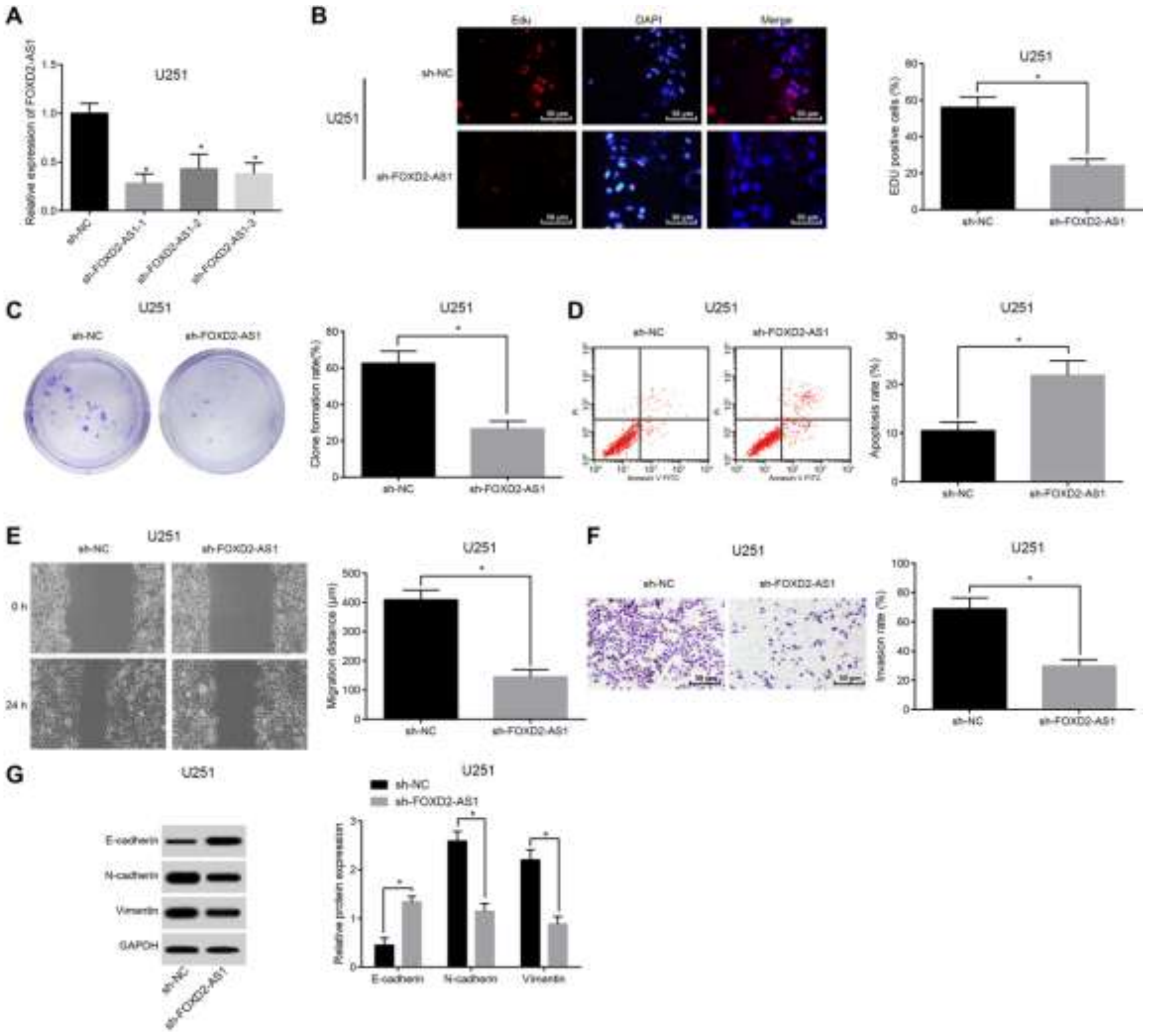

Supplementary Figure 1. FOXD2-AS1 silence overtly inhibits the proliferation, migration, invasion and EMT of U251 glioma cells, and promotes their apoptosis. (**A**) The expression of FOXD2-AS1 in U251 cells detected by RT-qPCR. (**B**) The proliferation activity in U251 cells detected by EdU assay; (**C**) The colony formation ability of U251 cells detected by colony formation assay; (**D**) The apoptosis of U251 cells in each group examined via flow cytometry; (**E**) The migration ability of U251 cells in each group detected by scratch test; (**F**) The invasion of U251 cells in each group tested via Transwell assay; (**G**) The expression of EMT-related factor tested via Western blot analysis. * $P < 0.05$, vs the sh-NC group; The data in the figure were all measurement data, represented by mean ± standard deviation. The comparison between the two groups was statistically analyzed by independent sample t test, and the experiment was repeated for three times.
